# Involvement of the agmatinergic system in the depressive-like phenotype of the *Crtc1* knockout mouse model of depression

**DOI:** 10.1038/tp.2016.116

**Published:** 2016-07-12

**Authors:** E M Meylan, L Breuillaud, T Seredenina, P J Magistretti, O Halfon, R Luthi-Carter, J-R Cardinaux

**Affiliations:** 1 Center for Psychiatric Neuroscience, Department of Psychiatry, University Medical Center, University of Lausanne, Prilly, Switzerland; 2 Service of Child and Adolescent Psychiatry, Department of Psychiatry, University Medical Center, University of Lausanne, Lausanne, Switzerland; 3 Laboratory of Functional Neurogenomics, Brain Mind Institute, Ecole Polytechnique Fédérale de Lausanne, Lausanne, Switzerland; 4 Division of Biological and Environmental Sciences and Engineering, King Abdullah University of Science and Technology, Thuwal, Saudi Arabia; 5 Laboratory of Neuroenergetics and Cellular Dynamics, Brain Mind Institute, Ecole Polytechnique Fédérale de Lausanne, Lausanne, Switzerland; 6 Department of Neuroscience, Psychology and Behaviour, University of Leicester, Leicester, UK

## Abstract

Recent studies implicate the arginine-decarboxylation product agmatine in mood regulation. Agmatine has antidepressant properties in rodent models of depression, and agmatinase (Agmat), the agmatine-degrading enzyme, is upregulated in the brains of mood disorder patients. We have previously shown that mice lacking CREB-regulated transcription coactivator 1 (CRTC1) associate behavioral and molecular depressive-like endophenotypes, as well as blunted responses to classical antidepressants. Here, the molecular basis of the behavioral phenotype of *Crtc1*^*−/−*^ mice was further examined using microarray gene expression profiling that revealed an upregulation of *Agmat* in the cortex of *Crtc1*^*−/−*^ mice. Quantitative polymerase chain reaction and western blot analyses confirmed *Agmat* upregulation in the *Crtc1*^*−/−*^ prefrontal cortex (PFC) and hippocampus, which were further demonstrated by confocal immunofluorescence microscopy to comprise an increased number of Agmat-expressing cells, notably parvalbumin- and somatostatin-positive interneurons. Acute agmatine and ketamine treatments comparably improved the depressive-like behavior of male and female *Crtc1*^*−/−*^ mice in the forced swim test, suggesting that exogenous agmatine has a rapid antidepressant effect through the compensation of agmatine deficit because of upregulated *Agmat*. Agmatine rapidly increased brain-derived neurotrophic factor (BDNF) levels only in the PFC of wild-type (WT) females, and decreased eukaryotic elongation factor 2 (eEF2) phosphorylation in the PFC of male and female WT mice, indicating that agmatine might be a fast-acting antidepressant with N-methyl-D-aspartate (NMDA) receptor antagonist properties. Collectively, these findings implicate Agmat in the depressive-like phenotype of *Crtc1*^*−/−*^ mice, refine current understanding of the agmatinergic system in the brain and highlight its putative role in major depression.

## Introduction

Major depressive disorder (MDD) is a complex neuropsychiatric disease comprising one of the leading causes of disability worldwide, with an estimated lifetime prevalence of 16%.^1^ However, the etiological mechanisms underlying MDD are not clearly established. Studies over the past decades have suggested that altered neuroplasticity is a cardinal feature of MDD,^2^ leading to the network hypothesis of depression. This latter proposes that impaired neuroplasticity related to problems in activity-dependent neuronal communication might alter information processing in the affected neural networks, and ultimately cause MDD.^3^ In line with this hypothesis, antidepressants have been shown to promote synaptogenesis, neurogenesis and dendritic growth in the hippocampus (HIP) of rodents.^4, 5^ These neurotrophic effects correlate with positive behavioral responses to antidepressants and are thought to rely, at least partly, on the activation of cAMP-response element-binding protein (CREB)-regulated genes, including increased signaling of the brain-derived neurotrophic factor (BDNF)-TrkB pathway.^4^ We and others have previously shown that CREB-dependent *Bdnf* expression requires CREB-regulated transcription coactivator 1 (CRTC1).^6, 7, 8^ CRTC1 has been shown to act as a neuronal calcium- and cAMP-sensitive coincidence detector and to promote CREB-dependent transcription.^6, 9^ In addition to its important role in *Bdnf* expression, CRTC1 has also been shown to be critical for specific aspects of neuroplasticity, as evidenced by its role in dendritic growth of developing cortical neurons^10, 11^ and its requirement for maintenance of long-term potentiation in the HIP.^6, 8^

To further understand the role and function of CRTC1, we generated a *Crtc1*-deficient mouse line.^12^ These mice present behavioral and molecular features mirroring mood disorders, such as increased behavioral despair, anhedonia, increased irritability/aggressiveness, decreased sexual motivation, social withdrawal, decreased dopamine and serotonin turnover in the prefrontal cortex (PFC), as well as decreased HIP and PFC expression in several neuroplasticity-related genes including *Bdnf* and its receptor *TrkB*.^13^ Furthermore, *Crtc1*^*−/−*^ mice exhibit a blunted antidepressant response to the selective serotonin reuptake inhibitor fluoxetine and to the tricyclic antidepressant desipramine in a behavioral despair paradigm.^13, 14^ Taken together, these findings suggest an important role for CRTC1 in the etiology of MDD and a possible involvement in treatment-resistant depression.

Substantial evidence supports the involvement of the arginine-decarboxylation product agmatine in MDD. This metabolite is widely expressed in several mammalian organs, including the brain. Agmatine synthesis competes with other arginine-dependent pathways, such as the urea cycle and nitric oxide (NO) synthesis.^15^ It is degraded by the enzyme agmatinase (Agmat) into putrescine, a key precursor for polyamine synthesis.^15, 16^ Accumulating evidence suggests that polyamines and their precursors have a role in the etiology and pathology of mental disorders, notably in mood disorders and suicidal behavior.^17, 18^ Agmatine has also been proposed to function as a neurotransmitter: it is stored in synaptic vesicles and released upon depolarization, followed by selective reuptake or degradation.^19^ In addition, agmatine has the ability to bind a wide range of receptors, including nicotinic receptors, imidazoline I_1_ and I_2_ receptors, α_2_-adrenergic receptors and serotoninergic 5HT-2A and 5HT-3 receptors.^20, 21, 22, 23^ Remarkably, agmatine also acts as a glutamate N-methyl-D-aspartate receptor (NMDAR) antagonist.^24^ This property is particularly interesting in light of the recent and growing interest in glutamate-based rapid-acting antidepressants, whose prototype is the NMDAR antagonist ketamine.^25, 26, 27, 28, 29^

Importantly, humans affected by depression show altered blood levels of agmatine, and post-mortem studies have shown increased Agmat levels in brain tissues from depressed individuals.^30, 31^ In rodents, cortical and hippocampal agmatine levels are decreased by restraint stress, and agmatine demonstrates neuroprotection against acute and chronic stress effects.^32, 33, 34, 35^ Furthermore, acute agmatine treatment has rapid antidepressant activities in depression-related paradigms such as the forced swim test (FST) and tail suspension test.^36^ These effects have been attributed to agmatine actions on monoaminergic and opioid systems, imidazoline and α_2_-adrenergic receptors, and NMDAR blockade.^36, 37, 38, 39^ Moreover, agmatine has the ability to modulate pro- and anti-oxidative balance in the HIP, which might also underlie its behavioral effects.^34^ Finally, a recent study has shown that in parallel to its antidepressant activity, agmatine increases HIP CREB phosphorylation and BDNF levels, and induces cell survival pathways.^40^ Altogether, these data suggest that agmatine stimulates several endogenous mood-regulating mechanisms known to be altered in MDD, leading to the conclusion that dysregulation of the agmatinergic system could play a role in the etiopathogenesis of MDD and agmatine supplementation might have a positive outcome on the disease.

In this study, we investigated the molecular basis for the depressive-like phenotype of *Crtc1*^*−/−*^ mice, which led us by differential expression analysis to discover a cortical upregulation of *Agmat* expression. Immunohistochemical studies revealed that mutant mice have an increased number of Agmat-expressing cells in the PFC and HIP, particularly parvalbumin (PV)- and somatostatin (Sst)-positive interneurons. Based on this result, we hypothesized that increased Agmat levels would result in reduced agmatine bioavailability in the brains of *Crtc1*^*−/−*^ mice, and that supplementation with exogenous agmatine would improve their depressive-like behavior. Indeed, we found that acute agmatine administration had a rapid antidepressant effect both in wild-type (WT) and *Crtc1*^*−/−*^ mice, the latter requiring a higher dose than WT, in accordance with their increased brain Agmat levels. Finally, we also investigated the molecular mechanisms underlying the rapid antidepressant effects of exogenous agmatine in WT and *Crtc1*^*−/−*^ mice. We found that agmatine induced BDNF translation in the PFC of WT mice, paralleled by dephosphorylation of eukaryotic elongation factor 2 (eEF2), suggesting NMDAR-mediated antidepressant mechanisms.

## Materials and methods

### Animals

*Crtc1*^*−/−*^ mice and WT littermates were generated and genotyped as previously described.^12^ Mice were housed under a 12-h light–dark cycle with *ad libitum* access to water and standard rodent chow diet. All animal experiments were conducted in accordance with the Swiss Federal Veterinary Office’s guidelines and were approved by the Cantonal Veterinary Service. All behavioral tests were carried out in the dark phase of the reverse light cycle according to the standard procedures. Male and female mice were weaned at 21 days and housed in same-sex sibling groups until being isolated at 5 weeks of age in order to avoid wounding of cage mates by aggressive *Crtc1*^*−/−*^ male mice.^13^ At the age of 8 weeks, animals were randomly assigned into treatment groups and either killed for molecular experiments or used for behavioral assessments.

### Brain microdissection

Male and female mice were killed by cervical dislocation, and the brain was rapidly placed in a stainless steel adult mouse brain slicer matrix with 1-mm coronal section slice intervals. A first cut included the PFC from which the olfactory bulbs and associated structures were removed. Total hippocampi were unrolled from the cortex. All the structures were sequentially quick-frozen in dry ice for mRNA and protein extraction and stored at −80 °C until further processing.

### Gene expression analysis

Total RNA was extracted and purified from the PFC and HIP using the RNAeasy Plus Minikit (Qiagen, Hombrechtikon, Switzerland) according to the manufacturers’ instructions. RNA concentrations were measured by Ultraviolet spectophotometry with a NanoDrop Lite (Thermo Scientific, Wilmington, DE, USA). Complementary DNA was prepared in a 50-μl reaction by reverse transcription, using 200 ng of RNA with Taqman Reagents and random hexamers (Applied Biosystems, Foster City, CA, USA). Complementary DNA (0.8 μl) was amplified on a 96-well plate using the SYBR Green PCR Master Mix (Applied Biosystem). Amplification was performed with an ABIPRISM 7500 real-time PCR system (Applied Biosystem). The program was 2 min at 50 °C, 10 min at 95 °C, followed by 45 cycles of 15 s at 95 °C and 1 min at 60 °C. Relative gene expression was quantified using the comparative *ΔΔ Ct* method and normalized with β-actin transcript levels.

The following primers were used at a concentration of 250 nm: *β-actin* forward 5′-GCTTCTTTGCAGCTCCTTCGT-3′, *β-actin* reverse 5′-ATATCGTCATCCATGGCGAAC-3′, *Agmat* forward 5′-TGGACAGCAAGCGAGTGGTACA-3′, *Agmat* reverse 5′-GGACCAGTGACTTCATCCAACAG-3′.

### Affymetrix gene expression arrays

Gene expression levels were evaluated using DNA microarrays (GeneChip Mouse Genome 430 version 2.0, Affymetrix, Santa Clara, CA, USA) and RNA from the cerebral cortices of female mice (*n*=5 for WT and *n*=5 for *Crtc1*^*−/−*^). Biotinylated cRNAs were prepared from 300 ng total RNA using the GeneChip 3′ IVT Express Kit (Affymetrix) following the manufacturer’s instructions. cRNA (15 μg) was hybridized to GeneChip arrays and processed, stained and scanned according to the manufacturer’s recommendations. The quality of input RNAs and cRNAs was verified with the Bioanalyzer 2100 (Agilent Technologies, Santa Clara, CA, USA) before use. Microarray quality control was performed using the software package provided on RACE.^41^ Chips with a median-normalized unscaled s.e. greater than 1.05 were excluded. Affymetrix annotations (version 3.0) were used for probeset-to-gene assignments. Mod *t*-statistics and false discovery rate corrections for multiple testing with a significance threshold of *P*<0.05 were used as criteria for differential expression, as described in Hochberg and Benjamini.^42^ Microarray data have been deposited in NCBI's Gene Expression Omnibus (GEO) and are accessible through GEO Series accession number GSE80633 (http://www.ncbi.nlm.nih.gov/geo/query/acc.cgi?acc=GSE80633).

### Western blot

PFC and HIP samples were manually homogenized with a microtube pestle in RIPA buffer (50 mM HEPES (pH 7.6), 150 mM NaCl, 1 mM EDTA (pH 7.5), 2.5 mM EGTA (pH 8.0), 10% glycerol, 1% NP-40, 1% deoxycholate, 0.1% SDS, with a protease inhibitor cocktail (Sigma, St Louis, MO, USA) and a phosphatase inhibitor cocktail (PhosSTOP, Roche, Rotkreuz, Switzerland) and extracted for 20 min at 4 °C. Protein quantification was performed with the Pierce BCA Protein Assay Kit (Thermo Scientific). Samples with low-protein extract (<2 mg ml^−1^) were excluded. Fifty μg of tissue homogenates were diluted 1:1 with sample buffer (50 mM Tris-HCl (pH 6.8), 100 mM dithiothreitol, 2% SDS, 9% glycerol, 1% bromophenol blue), separated on a 12% SDS-polyacrylamide gel and proteins were transferred to polyvinylidene difluoride membranes with a semi-dry blotting system (Bio-Rad, Hercules, CA, USA). Blots were blocked for 1 h at room temperature (RT) in TBST (10 mM Tris-HCl (pH 7.4), 150 mM NaCl, 0.1% Tween-20), supplemented with 5% skim milk powder. Blots were subsequently incubated with a primary antibody in TBST plus 5% bovine serum albumin overnight at 4 °C. Finally, polyvinylidene difluoride membranes were incubated for 1 h at RT with horseradish peroxidase (HRP)-conjugated secondary antibodies in TBST plus 5% skim milk powder, and were developed using a Pierce ECL Western Chemiluminescence Detection Kit (Thermo Scientific). The following antibodies and dilutions were used: rabbit α-Agmat 1:400 (sc-98802, Santa Cruz Biotechnology, Dallas, TX, USA), rabbit α-BDNF 1:500 (sc-546, Santa Cruz Biotechnology), rabbit α-phospho-eEF2 1:1000 (#2331, Cell Signaling, Danvers, MA, USA), rabbit α-eEF2 1:1000 (#2332, Cell Signaling), mouse α-β-actin 1:10 000 (Ab-6276, Abcam, Cambridge, UK), donkey HRP-α-rabbit 1:2000 (#NA934, GE Healthcare, Little Chalfont, UK) and sheep HRP-α-mouse 1:2000 (#NA931, GE Healthcare). Quantification of band intensity was performed with the Image J software (National Institute of Health, Bethesda, MD, USA). Agmat and BDNF band intensities were normalized with β-actin signals; phospho-eEF2 band intensities were normalized with total eEF2 signals.

### Immunofluorescence

Eight-week-old male mice were deeply anesthetized using sodium pentobarbital and intracardially perfused with saline followed by 4% buffered paraformaldehyde. Brains were dissected out, postfixed for 1 h in 4% paraformaldehyde and cryoprotected in 30% sucrose. Brain sections of 35 μm were cut with a freezing microtome (Microm, Thermo Fisher Scientific, Waltham, MA, USA) and stored at −20 °C in a cryoprotectant solution. Blocking (1 h, RT) as well as primary (overnight, 4 °C) and secondary antibody incubation (1 h, RT) were performed in phosphate-buffered saline+0.3% Triton X-100+2% normal horse serum+0.2% bovine serum albumin. Slices were washed three times in phosphate-buffered saline+0.3% Triton X-100 after each incubation. The following antibodies and dilutions were used: rabbit α-Agmat 1:100 (sc-98802, Santa Cruz Biotechnology), mouse α-Parvalbumin 1:2500 (PV235, Swant, Marly, Switzerland), mouse α-Calretinin 1:2500 (CR7697, Swant), goat α-Somatostatin 1:500 (sc-7819, Santa Cruz Biotechnology), Cy3-conjugated donkey α-rabbit 1:500 (#711-165-152, Jackson Immunoresearch, West Grove, PA, USA), Alexa Fluor 488-conjugated goat α-mouse 1:500 (A-21121, Molecular Probes, Eugene, OR, USA) and Alexa Fluor 488-conjugated donkey α-goat 1:500 (A11055, Invitrogen, Carlsbad, CA, USA). After the secondary antibody incubation, slices were washed, stained with 4,6-diamidino-2-phenylindole 1:30 000 (Invitrogen), mounted on glass slides with the antifade Vectashield medium (Vector Laboratories, Burlingame, CA, USA) and analyzed with a Zeiss LSM 710 Quasar Confocal Microscope (Carl Zeiss, Oberkochen, Germany). Image processing and cell counting were carried out with the Image J Software (National Institute of Health).

### Agmatine and ketamine treatment

Agmatine sulphate salt and ketamine (Ketanarkon) were, respectively, purchased from Sigma and Streuli Pharma (Uznach, Switzerland) and dissolved in saline solution. Male and female mice were intraperitoneally injected with 10 ml kg^−1^ of agmatine (10 or 50 mg kg^−1^) or ketamine (3 mg kg^−1^). Controls were injected with saline. Injections were performed 30 min before the FST for two consecutive days.

### FST

A 2-day test was performed (days 1 and 2). Mice were put during 5 min in a 5-l glass beaker (26 cm tall, ø18 cm) filled to a depth of 22 cm with tap water (25±1 °C). Sessions were videotaped from above and manually analyzed non-blindly by the experimenter with the Ethovision 3.1 Software (Noldus Information Technology, Wageningen, The Netherlands) for immobility and climbing time. Mice were judged immobile when no detectable movement was observed, except for minor movements to keep their head above the water. The experiment was conducted in a room with a light intensity of ~35 lux. Immediately after the test on day 2, mice were killed for BDNF and phospho-eEF2 measurements.

### Statistical analyses

The number of animals tested in each group is specified in the figure legends. Owing to the small number of WT and *Crtc1*^*−/−*^ mice per litter and a limited breeding cage space, all experiments were performed sequentially with several batches of mice, and their data were combined. Sample sizes were determined based on power analysis and common practice in behavioral experiments (~10 animals per group). Statistical analyses (other than those employed for microarray analyses (see above)) were performed using the Statistica 8.0 Software (StatSoft, Tulsa, OK, USA). All data are presented as mean±s.e.m. *P*<0.05 were considered statistically significant. A Shapiro–Wilk test and a Levene test were first performed to assess data normality and variance homogeneity. All results were found to follow normal distribution and to display similar variance. For immunofluorescence, quantitative PCR and western blot data, a two-tailed Student's *t*-test was performed when only two groups were compared (WT versus *Crtc1*^*−/−*^ mice). For behavioral data, BDNF and phospho-eEF2 data, a two-way analysis of variance (ANOVA; with genotype and treatment as independent variables) was performed, followed by a Fisher's Least Significant Difference (LSD) *post hoc* test.

## Results

### Male and female *Crtc1*^*−/−*^ mice exhibit increased levels of *Agmat* mRNA and protein in the PFC and HIP

**Figure 1:**
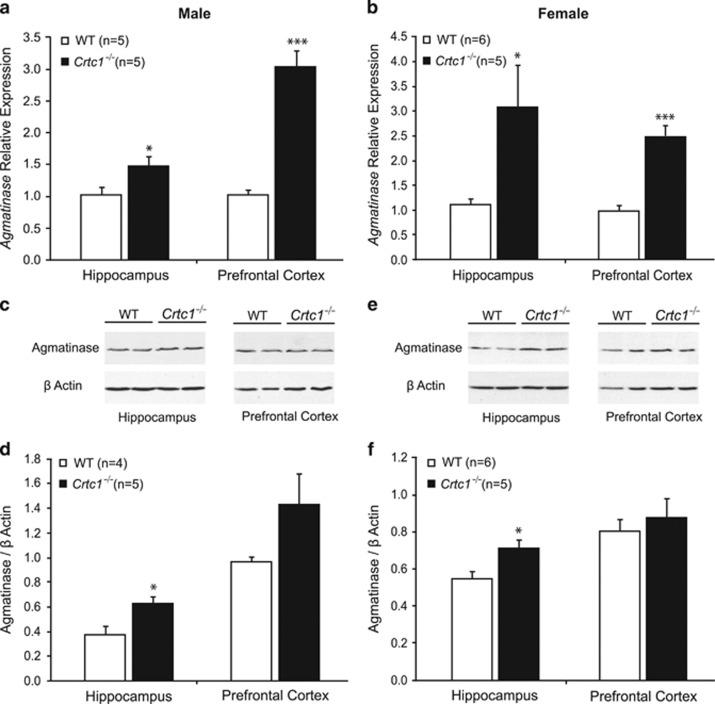
Increased levels of agmatinase (Agmat) in *Crtc1*^*−/−*^ mice. (**a**) Real-time quantitative PCR measurements showed an increased expression of *Agmat* in the hippocampus (HIP) and prefrontal cortex (PFC) of male *Crtc1*^*−/−*^ mice (*n*=5) compared with wild-type (WT) littermates (*n*=5). (**b**) *Agmat* was also overexpressed in the HIP and PFC of female *Crtc1*^*−/−*^ mice (*n*=6) compared with WT control mice (*n*=5). Representative western blot of Agmat and β-actin is shown in **c** for male mice and in **e** for female mice. Quantitative analyses of western blot showed increased protein levels of Agmat in the HIP of male *Crtc1*^*−/−*^ mice (*n*=4) compared with WT mice (*n*=5**; d**). Protein levels of Agmat were also increased in the HIP of female *Crtc1*^*−/−*^ mice (*n*=6) compared with WT mice (*n*=5; **f**). Results are presented as ratio between Agmat and β-actin signals. Data are mean±s.e.m. *P<0.5, ***P<0.001.

To identify gene expression changes associated with *Crtc1* deficiency, we performed genome-wide transcriptomic profiling analyses of cortical samples from *Crtc1*^*−/−*^ and WT female mice using oligonucleotide microarrays (Table 1). Among the downregulated genes were CREB target genes that we previously showed to have a decreased expression in the PFC and HIP of *Crtc1*^*−/−*^ male mice.^13^ Interestingly, a few genes were upregulated in *Crtc1*^*−/−*^ mice, and amidst them *Agmat*, whose expression was increased by 1.67-fold. To follow up and confirm this finding, we measured *Agmat* messenger RNA (mRNA) and protein levels in male and female *Crtc1*^*−/−*^ mice (Figure 1). We focused our investigations on the PFC and HIP, as these two regions are widely implicated in mood disorders and are known to have high levels of agmatine.^43^ Quantitative PCR analyses of *Agmat* mRNA levels found a significant (*t*=−3.31, degree of freedom (df)=9, *P*=0.013) 1.5-fold increase of *Agmat* mRNA in the HIP and a threefold increase (*t*=−6.72, df=8, *P*<0.001) in the PFC of male *Crtc1*^*−/−*^ mice (Figure 1a). In female *Crtc1*^*−/−*^ mice, a significant (*t*=−2.65, df=9, *P*=0.029) threefold increase in *Agmat* mRNA was observed in the HIP and a 2.5-fold increase (*t*=5.45, df=9, *P*<0.001) in the PFC (Figure 1b). Western blot analysis of extracts from these same structures showed an Agmat protein band at the expected size of ~35 kDa (Figures 1c and e), which was subsequently quantified (normalized to β-actin signal). *Crtc1*^*−/−*^ male mice had increased levels of Agmat protein in the HIP (*t*=−2.72, df=6, *P*=0.036), whereas a nonsignificant trend (*t*=−1.51, df=9, *P*=0.152) of increased Agmat was observed in the PFC (Figure 1d). In female *Crtc1*^*−/−*^ mice, a similar increase was found in the HIP (*t*=−2.58, df=9, *P*=0.027), whereas no change in Agmat protein content could be seen in the PFC (*t*=−0.61, df=9, *P*=0.553; Figure 1f). Taken together, these results confirm an upregulation of *Agmat* gene expression in the HIP and PFC of *Crtc1*^*−/−*^ mice, independently of gender. Although Agmat protein levels only partially correlated with the gene expression data (which might reflect translational regulation or a complex subcellular protein localization), these results strongly suggest that *Crtc1*^*−/−*^ mice have an altered agmatinergic system.

### *Crtc1*^*−/−*^ mice have an increased number of Agmat-expressing cells in the PFC and in several regions of the HIP

**Figure 2:**
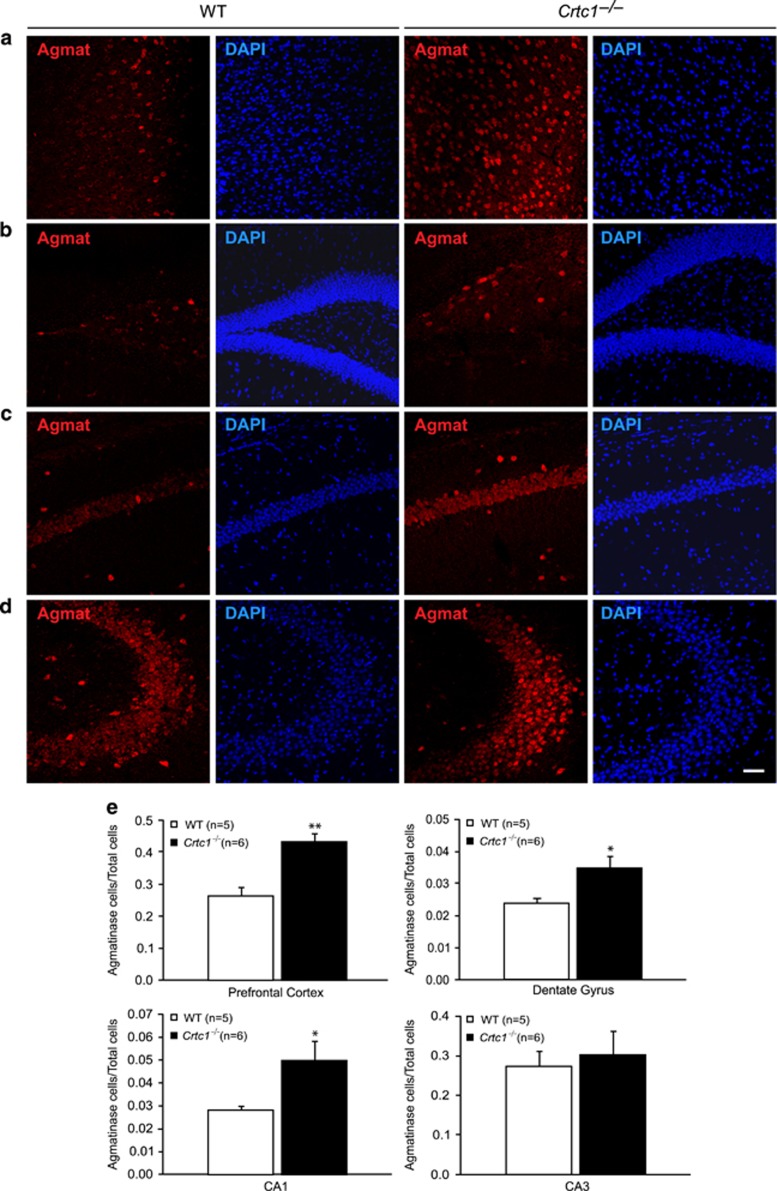
Increased number of agmatinase (Agmat)-expressing cells in male *Crtc1*^*−/−*^ mice. Representative immunofluorescence staining of Agmat-expressing cells (red) and total cells (4,6-diamidino-2-phenylindole (DAPI) staining) in the prefrontal cortex (PFC; **a**), and in the dentate gyrus (DG; **b**), CA1 (**c**) and CA3 (**d**) regions of the hippocampus (HIP). Cell counting resulted in an increased number of Agmat-expressing cells in the PFC, DG and CA1 regions of male *Crtc1*^*−/−*^ mice (*n*=6) compared with wild-type (WT) littermates (*n*=5; **e**). No difference in number of Agmat-expressing cells was found in the CA3 region. Results are expressed as ratio between number of Agmat-expressing cells and total number of cells. Data are mean ±s.e.m. *P<0.5, **P<0.01. Scale bar, 50 μm.

To determine whether the increased *Agmat* expression in *Crtc1*^*−/−*^ mice was because of higher Agmat levels or an increased number of Agmat-expressing cells, we visualized Agmat protein expression using immunofluorescence in the PFC and in the CA1, CA3 and dentate gyrus (DG) subregions of the HIP (Figure 2). Staining revealed numerous cells in the PFC (Figure 2a), and in the DG (Figure 2b), CA1 (Figure 2c) and CA3 (Figure 2d) regions of the HIP, and its subcellular localization appeared mainly perinuclear within those structures. In the HIP, staining could be observed in the pyramidal cell layer, but appeared stronger in interneuron-like cells. Agmat-expressing cells were counted and results were normalized to total numbers of cells, counted with 4,6-diamidino-2-phenylindole staining (Figure 2e). Agmat-positive cells counting revealed a significant increased number of cells in the PFC of *Crtc1*^*−/−*^ mice (+60%, *t*=−4.23, df=9, *P*=0.001) as compared with WT littermates. This could also be observed in the DG (+45%, *t*=−2.55, df=9, *P*=0.029) and CA1 regions of the HIP (+70%, *t*=−2.28, df=9, *P*=0.049). No differences in the numbers of Agmat-expressing cells could be seen in the CA3 region of the HIP (*t*=−0.40, df=9, *P*=0.698). Overall, these data suggest that the increased Agmat expression found in *Crtc1*^*−/−*^ mice would be the result of a higher number of Agmat-expressing cells in the PFC and selected HIP subregions. Moreover, the morphology and localization of the hippocampal cells expressing higher Agmat levels indicated that they could be GABAergic interneurons.

### Characterization of Agmat-expressing cells in the PFC and HIP

**Figure 3:**
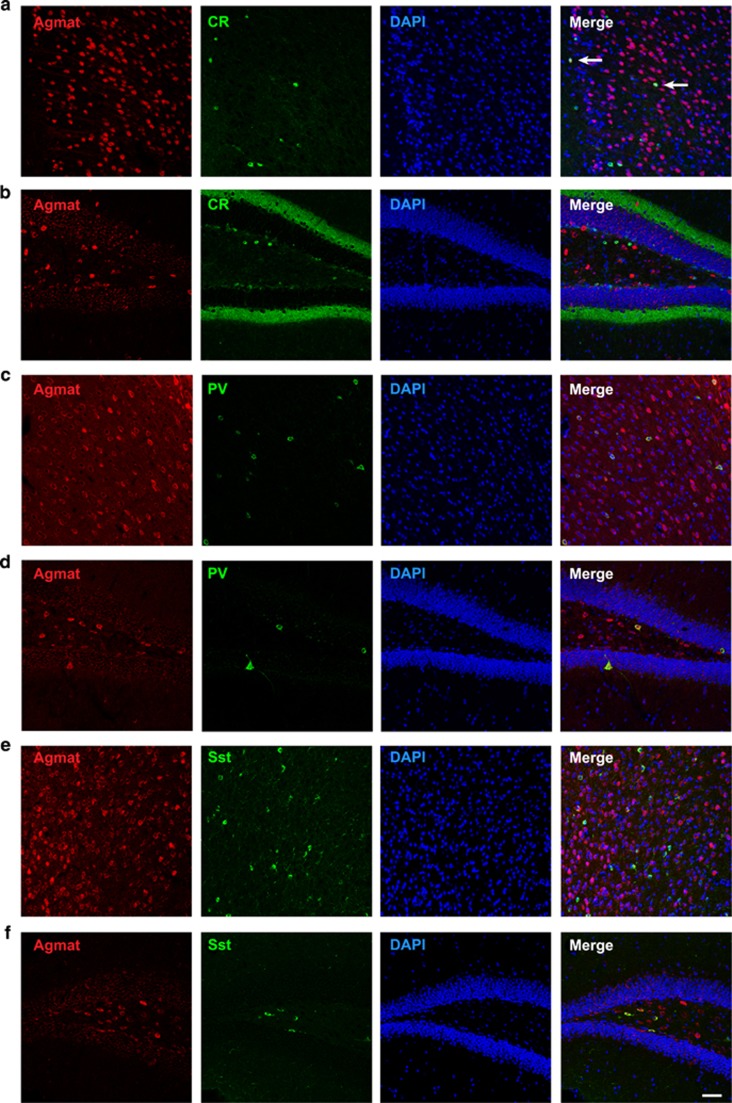
Characterization of GABAergic interneuron subpopulations expressing agmatinase (Agmat) in the prefrontal cortex (PFC) and hippocampus (HIP) of wild-type (WT) male mice. Double immunofluorescence labeling of (**a**, **b**) Agmat and calretinin (CR; **c,** **d**) Agmat and parvalbumin (PV), and (**e**, **f**) Agmat and somatostatin (Sst) in (**a**, **c**, **e**) PFC and (**b**, **d**, **f**) dentate gyrus (DG) of the HIP. Total cells were identified by nuclear 4,6-diamidino-2-phenylindole (DAPI) staining. Merged images showed few colocalization of Agmat and CR staining in the PFC (**a**) as indicated by arrows. No colocalization could be observed in the DG (**b**). All PV and Sst interneurons also expressed Agmat in the PFC (**c**, **e**) and DG (**d**, **f**). Scale bar, 50 μm.

As hippocampal Agmat-expressing cells have an interneuron-like morphology, we characterized Agmat-expressing cells with markers for specific GABAergic interneuron types. Bernstein *et al.*^44^ studied regional and cellular expression of *Agmat* in the rat brain and found that Agmat colocalized with calretinin (CR)-expressing interneurons in the cortex and CA1 region of the HIP.^44^ We therefore performed double immunolabeling of CR and Agmat in the PFC and HIP of WT mice (Figure 3 and Supplementary Figure S1). In the PFC, we observed very little colocalization between CR and Agmat staining (Figure 3a, double-labeled cells indicated with arrows). Little colocalization was also seen in the CA3 region of the HIP (Figure S1b). Moreover, no colocalization was observed in the DG and CA1 regions of the HIP (Figure 3b and Supplementary Figure S1a, respectively). In order to further investigate which type of interneurons expressed Agmat, we performed double immunostaining of Agmat- and PV-expressing cells. We observed high colocalization of Agmat and PV cells in all studied structures: PFC, DG, CA1 and CA3 regions of the HIP (Figures 3c and d and Supplementary Figure S1c and d, respectively). Indeed, nearly all PV-expressing cells were also Agmat-positive. On the other hand, there were some Agmat-expressing cells that were not PV-positive, therefore suggesting that other types of cells express Agmat.

Several lines of evidence involved *somatostatin* (*Sst*) in mood disorders,^45^ and we previously observed that *Sst* was downregulated in the brain of *Crtc1*^*−/−*^ mice.^13^ Therefore, we investigated whether *Agmat* might be expressed in somatostatinergic interneurons by performing double immunostaining of Agmat- and Sst-expressing cells. As for PV staining, most of Sst-expressing cells were colocalized with Agmat in all regions observed: PFC, DG, CA1 and CA3 regions of the HIP (Figures 3e and f and Supplementary Figure S1e and f, respectively). Colocalization was, however, less extended in the PFC; as some Sst-expressing cells were not colocalized with Agmat staining.

Altogether, these data confirm the expression of Agmat in specific GABAergic interneuron subpopulations, with apparent high expression in PV and Sst interneurons, and slight colocalization with CR interneurons.

### Rapid ketamine-like antidepressant effect of acute agmatine in male and female WT and *Crtc1*^*−/−*^ mice

**Figure 4:**
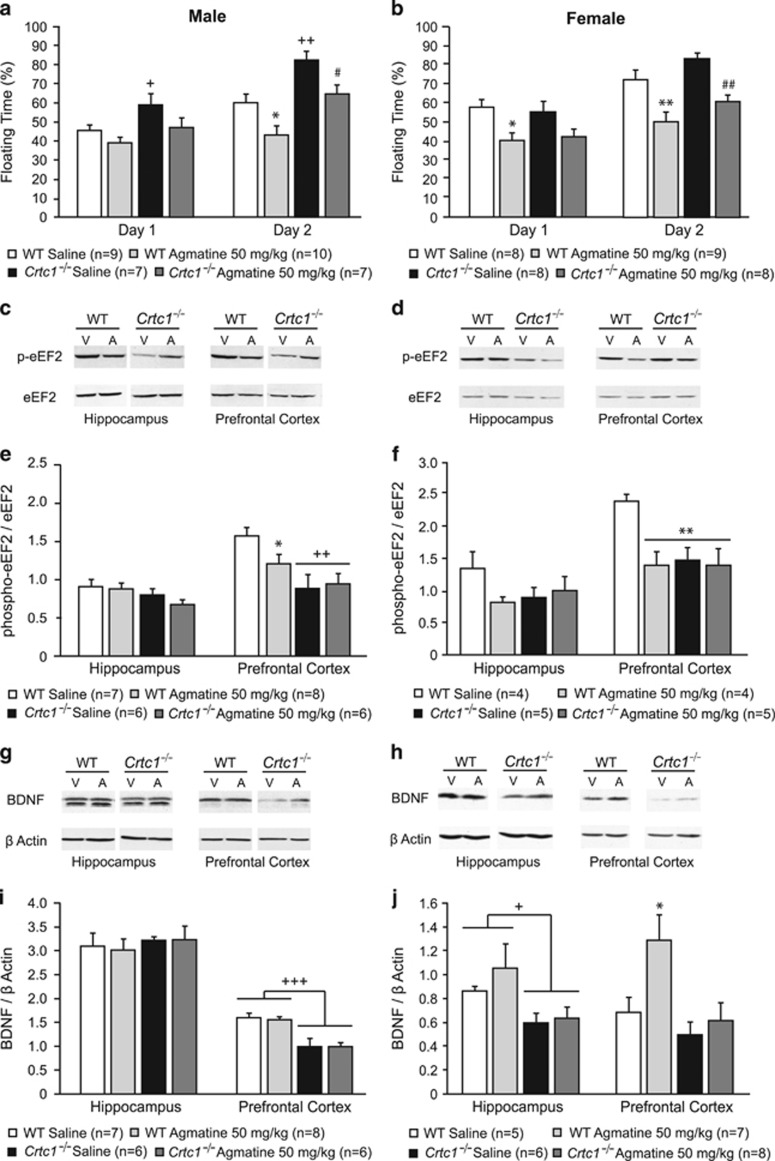
Behavioral and molecular effects of agmatine treatment on male and female wild-type (WT) and *Crtc1*^*−/−*^ mice. (**a,** **b**) Effects of acute agmatine treatment (50 mg kg^−1^, intraperitoneal (IP)) 30 min before a forced swim test (FST) during two consecutive days. In male mice (**a**) vehicle-treated *Crtc1*^*−/−*^ mice (*n*=7) showed higher immobility levels than vehicle-treated WT mice (*n*=9) on both days of test (^+^*P*<0.05, ^++^*P*<0.01). On Day 2, agmatine significantly decreased the immobility time of WT mice (*P<0.05) and *Crtc1*^*−/−*^ mice (^#^*P*<0.05; *n*=10 and *n*=7, respectively). In female mice (**b**) WT mice treated with agmatine (*n*=8) showed significantly decreased immobility time (*P<0.05, **P<0.01) compared with vehicle-treated WT mice (*n*=9) on both days of test. On day 2, agmatine-treated *Crtc1*^*−/−*^ mice (*n*=8) also presented significantly decreased immobility time (^##^*P*<0.01) than vehicle-treated *Crtc1*^*−/−*^ mice (*n*=8). (**c**–**f**) Effects of acute agmatine treatment (50 mg kg^−1^, IP) on eukaryotic elongation factor 2 (eEF2) phosphorylation. (**c**, **d**) A representative western blot for phospho-eEF2 and total eEF2 in the hippocampus (HIP) and prefrontal cortex (PFC) of WT and *Crtc1*^*−/−*^ mice (V, vehicle-treated; A, agmatine-treated) in male (**c**) and female (**d**) mice. (**e**, **f**) Quantitative analyses of p-eEF2 western blot in male (**e**) and female (**f**) mice. In male mice, no effect of agmatine could be seen in the HIP of WT and *Crtc1*^*−/−*^ mice. In the PFC, agmatine-treated WT mice (*n*=8) presented lower levels of p-eEF2 than vehicle-treated WT mice (*n*=7; *P<0.05). Both vehicle- and agmatine-treated *Crtc1*^*−/−*^ mice (*n*=6 for both) displayed lower p-eEF2 levels than WT mice (^++^*P*<0.01); agmatine treatment had no effect on p-eEF2 levels in these animals (**e**). In female mice, quantification showed no effect of agmatine treatment in the HIP of WT and *Crtc1*^*−/−*^ mice (**f**). In the PFC, agmatine-treated WT mice (*n*=4) presented lower levels of eEF2 phosphorylation compared with vehicle-treated WT animals (*n*=4; **P<0.01). *Crtc1*^*−/−*^ mice treated with vehicle (*n*=5) or agmatine (*n*=5) also displayed lower levels of eEF2 phosphorylation than WT mice (**P<0.01). Agmatine treatment had no effect on *Crtc1*^*−/−*^ mice. Results are presented as ratio between phospho-eEF2 and total eEF2 signals. (**g**–**j**) Effects of acute agmatine treatment (50 mg kg^−1^, IP) on brain-derived neurotrophic factor (BDNF) protein level. (**g,** **h**) A representative western blot for BDNF and β-actin in the HIP and PFC of WT and *Crtc1*^*−/−*^ mice in male (**g**) and female (**h**) mice. (**i, j**) Quantitative analyses of BDNF western blot in male (**i**) and female (**j**) mice. In male mice, no effect of agmatine could be seen in the HIP of WT and *Crtc1*^*−/−*^ mice. In the PFC, both vehicle- and agmatine-treated *Crtc1*^*−/−*^ mice (*n*=6 for both) displayed lower BDNF levels than vehicle- and agmatine-treated WT mice (*n*=7 and *n*=8, respectively; ^+++^*P*<0.001) (**i**). In female mice, quantitative analyses of western blot showed decreased levels of BDNF in the HIP of all *Crtc1*^*−/−*^ animals (*n*=14) compared with WT animals (*n*=13), independently of the treatment (^+^*P*<0.05). In the PFC, agmatine-treated WT mice (*n*=7) presented higher levels of BDNF compared with vehicle-treated WT animals (*n*=5; *P<0.05). *Crtc1*^*−/−*^ mice treated with agmatine (*n*=8) did not present different BDNF levels than those treated with saline (*n*=6; **j**). Results are presented as ratio between BDNF and β-actin signals. Data are mean±s.e.m.

Given the possible role of an agmatine deficit in depression and the observed increase in Agmat levels in the brain of *Crtc1*^*−/−*^ mice, we postulated that their depressive-like behavior is due, at least in part, to a dysregulated agmatinergic system. To test this hypothesis, we treated WT and *Crtc1*^*−/−*^ mice with acute intraperitoneal (IP) injections of agmatine and tested antidepressant effects in the FST, a classical test for rodent depression-related behavior. We hypothesized that restoring agmatine levels by exogenous supplementation would normalize behavioral response to the helplessness-inducing effects of FST. We first treated the animals with agmatine at 10 mg kg^−1^ and compared their depressive-like behavior in the FST with saline-injected control animals. This protocol was repeated a second time on the next day. The antidepressant effects of agmatine were assessed by measuring the floating (immobility) time of the mice. Agmatine had a significant antidepressant effect on WT mice but failed to significantly reduce *Crtc1*^*−/−*^ mouse immobility time (data not shown). However, a tendency to decrease the immobility time of *Crtc1*^*−/−*^ mice prompted us to repeat this experiment with an increased dose of agmatine (50 mg kg^−1^, IP; Figures 4a and b). For male mice (Figure 4a), a significant effect of genotype on immobility could be seen on both days of test, as shown by two-way ANOVA (Day 1: F_(1,29)_=6.74, *P*=0.014; Day 2: F_(1,29)_=19.4, *P*<0.001). *Post hoc* analyses revealed that vehicle-treated *Crtc1*^*−/−*^ mice presented higher immobility time than WT mice (Day 1: +13%, *P*=0.031, Day 2: +22%, *P*=0.004). A significant treatment effect could be observed on the second day of test (F_(1,29)_=12.38, *P*=0.001) as agmatine significantly decreased the immobility time of WT and *Crtc1*^*−/−*^ mice (*P*=0.012 and *P*=0.026, respectively). For female mice (Figure 4b), the analysis of immobility time by two-way ANOVA revealed a significant treatment effects for both days of test (Day 1: F_(1,28)_=10.28, *P*=0.003; Day 2: F_(1,28)_=23.46, *P*<0.001). *Post hoc* analyses showed that agmatine significantly reduced immobility time in WT female mice on day 1 (−30%, *P*=0.014) and of both genotypes on day 2 (WT: −30%, *P*=0.003; *Crtc1*^*−/−*^: −30%, *P*=0.001). Thus, 50 mg kg^−1^of agmatine reduced the immobility time of male and female *Crtc1*^*−/−*^ mice. Overall, these data confirm the antidepressant effect of an acute agmatine treatment in WT animals. *Crtc1*^*−/−*^ mice also respond to the antidepressant effect of agmatine, but require a higher dose (50 mg kg^−1^).

Ketamine and other NMDAR antagonists have been shown to have rapid and long-lasting antidepressant effects in behavioral despair paradigms such as the FST.^27^ As agmatine also acts as a NMDAR antagonist,^24^ we assessed the antidepressant effect of ketamine in male and female WT and *Crtc1*^*−/−*^ mice (Supplementary Figure S2). Interestingly, ketamine (3 mg kg^−1^, IP) significantly decreased the depressive-like behavior of WT and *Crtc1*^*−/−*^ mice of both sexes in a very similar way as agmatine did, which suggested that the rapid antidepressant action of agmatine and ketamine may involve the same molecular pathways.

### Characterization of pathways involved in agmatine antidepressant effect

Although agmatine antidepressant effects have been established, the underlying molecular mechanisms remain unclear. A recent study suggested that chronic agmatine treatment induces an increase of BDNF protein levels, as well as an increased phosphorylation of CREB, PKA and other kinases involved in pathways associated with neuroplasticity.^40^ Although the underlying cellular and molecular mechanisms of ketamine’s antidepressant action are not completely understood, they involve the rapid induction of BDNF translation via activation of the mammalian target of rapamycin (mTOR) pathway as seen by dephosphorylation of eEF2.^27^ Therefore, we investigated whether agmatine’s rapid antidepressant effect involved NMDAR blockade-associated changes in BDNF levels and eEF2 phosphorylation. We measured the levels of phospho-eEF2 and BDNF proteins in the PFC and HIP of male and female mice treated with 50 mg kg^−1^ (Figures 4c and j). Western blot for phospho-eEF2 and total eEF2 showed a single band at the expected size of 95 kDa (Figures 4c and d). Phospho-eEF2 signal was quantified and normalized over total eEF2 signal. In male mice (Figure 4e), a two-way ANOVA showed no effect of genotype or treatment in the HIP. A significant effect of genotype could be seen in the PFC (F_(1,23)_=13.34, *P*=0.001) as both groups of *Crtc1*^*−/−*^ mice presented lower levels of phospho-eEF2 (*P*=0.001 and *P*=0.002). Agmatine treatment had no effect on p-eEF2 levels of *Crtc1*^*−/−*^ mice, but it significantly reduce p-eEF2 in WT mice (*P*=0.04). In female mice (Figure 4f), a two-way ANOVA showed no effect of genotype or treatment in the HIP. A trend to decreased levels of phosphorylated eEF2 was present in agmatine-treated WT mice and in both groups of *Crtc1*^*−/−*^ animals. In the PFC, significant effects of genotype (F_(1,13)_, *P*=0.011), treatment (F_(1,13)_, *P*=0.043) and genotype × treatment (F_(1,13)_, *P*=0.011) were observed. *Post hoc* analyses revealed that agmatine significantly decreased the phosphorylation of eEF2 in WT mice (*P*=0.003). Both groups of *Crtc1*^*−/−*^ mice also presented decreased levels of phospho-eEF2 as compared with vehicle-treated WT mice (*Crtc1*^*−/−*^ Vehicle: *P*=0.001; *Crtc1*^*−/−*^ Agmatine: *P*=0.002). Agmatine had no effect on the phosphorylation of eEF2 in *Crtc1*^*−/−*^ mice.

Western blot for BDNF revealed a single band at the expected size of 14 kDa (Figures 4g–h). BDNF signal was quantified and normalized with β-actin signal. In male mice (Figure 4i), no effect of genotype or treatment could be observed in the HIP. In the PFC, a two-way ANOVA showed an effect of genotype (F_(1, 23)_=29.12, *P*<0.001). *Post hoc* analysis revealed that both vehicle- and agmatine-treated *Crtc1*^*−/−*^ mice presented lower levels of BDNF protein than WT mice (*P*<0.001 for both groups). Agmatine treatment had no effect on BDNF levels of WT and *Crtc1*^*−/−*^ mice. In female mice (Figure 4j), a two-way ANOVA revealed a significant effect of genotype in both structures (PFC: F_(1,22)_=6.78, *P*=0.016; HIP: F_(1,22)_=6.27, *P*=0.02). *Post hoc* analyses showed that both groups of *Crtc1*^*−/−*^ mice had significantly lower levels of BDNF than WT animals in the HIP, independently of the treatment (*P*=0.01). No effect of agmatine treatment could be seen in the HIP, whereas it was significant in the PFC (F_(1,22)_=4.63, *P*=0.042). *Post hoc* analyses indicated that agmatine significantly increased BDNF protein levels in the PFC, however, only in WT mice (*P*=0.024). Taken together, these results provide evidence that agmatine induces eEF2 dephosphorylation in WT male and female mice, thus suggesting activation of the mTOR pathway, through its NMDAR antagonist property. Agmatine effects on BDNF levels are gender-dependent, as agmatine rapidly induces BDNF translation in WT female mice, but not in WT male mice.

## Discussion

In this study, we showed for we believe the first time a link between downregulation of the CRTC1–CREB pathway and alteration of the agmatinergic system in the context of a rodent model of depression. We found that CRTC1-deficient mice exhibit increased mRNA and protein levels of Agmat in the HIP and PFC. We also determined that these higher Agmat levels are mainly because of an increased number of *Agmat*-expressing cells in the PFC and HIP of *Crtc1*^*−/−*^ mice. These findings suggest that *Crtc1*^*−/−*^ mice have a dysregulated agmatinergic system resulting in increased *Agmat* expression and ensuing decreased agmatine levels. Hence, the depressive-like phenotype of these animals would be in keeping with the protective and antidepressant role of endogenous agmatine.

Immunofluorescent detection of Agmat revealed that this enzyme is mainly expressed in interneurons in accordance with a previous characterization in the rat brain.^44^ As *Crtc1*^*−/−*^ mice have more *Agmat*-expressing cells, these mice might also have overall GABAergic system alteration. In line with this, agmatine and GABA seem to be closely related because agmatine is degraded into putrescine, whose derived polyamines can be used as GABA precursors.^16^ Therefore, we hypothesize that dysregulation of agmatine metabolism might lead to abnormal GABA regulation and ultimately to overall impaired interneuronal circuitry.

A characterization of the subpopulations of GABAergic interneurons expressing *Agmat* showed that it mainly colocalizes with PV and Sst interneurons, whereas no or little colocalization with CR interneurons was observed. These findings are in contradiction with the study of Bernstein *et al.*,^44^ which showed that Agmat was mainly found in CR interneurons. The reason for this discrepancy is unclear. Future studies should focus on a deeper characterization of *Agmat*-expressing cells, as Agmat staining revealed that it is present in many cells in the mouse brain and thus probably in a wide range of cell types.

The depressive-like behavior of *Crtc1*^*−/−*^ mice was successfully normalized by acute agmatine treatment as efficiently as ketamine’s effect. This suggests that their altered agmatinergic system contributes to their phenotype. It is noteworthy that only a higher dose of agmatine (50 mg kg^−1^) was effective, suggesting that the 10 mg kg^−1^ dose was not sufficient to compensate for a possible decrease in agmatine levels and to restore normal agmatinergic functions.

When looking at the molecular effects of agmatine, we found that it was able to induce an increase in BDNF protein levels in the PFC of WT females. This agmatine-induced BDNF upregulation was paralleled by eEF2 dephosphorylation, which stimulates protein translation. This mechanism has been shown to underlie the rapid antidepressant effect of NMDAR antagonists such as ketamine and MK-801.^26, 27, 46^ Therefore, our results suggest that agmatine acts as an antidepressant, possibly through this pathway. This is in line with the ability of agmatine to block NMDAR and the involvement of this function in its antidepressant effects.^36, 47, 48^ In contrast, the behavioral effects of agmatine in WT males were apparently not mediated by BDNF because agmatine did not increase its levels in HIP and PFC. The mechanisms that underlie these sex differences are still unclear, but not completely unexpected. These gender-specific effects are actually of much interest in the light of the female preponderance in major depression. Sex differences have been reported in animal models of depression. For instance, the impact of BDNF signaling on depression-like behavior is different in male and female mice.^49^ Moreover, it has been shown that hippocampal NO may contribute to sex difference in depressive-like behaviors.^50^ This study showed that stress promotes hippocampal NO production in male mice, whereas stress suppresses it in female ones. Worthy of note, both NO excess in male mice and shortage in female mice resulted in depressive-like behaviors through affecting CREB activation.

Interestingly, the effects of agmatine on BDNF and eEF2 were restricted to WT animals. *Crtc1*^*−/−*^ male and female mice displayed basal lower levels of phospho-eEF2 in the PFC, and agmatine did not decrease them further nor did it increase BDNF levels, which suggest a dysregulation of this pathway and the involvement of alternative mechanisms underlying agmatine’s antidepressant effects in these animals.

In conclusion, our results provide evidence for the involvement of the agmatinergic system in the *Crtc1*^*−/−*^ mouse model of depression, and lend support to previous reports of the antidepressant properties of agmatine. The comparable rapid antidepressant effects of agmatine and ketamine in WT and *Crtc1*^*−/−*^ mice, as well as the molecular effects that acute agmatine treatment causes in the brain of WT mice suggest that agmatine possibly functions as a fast-acting antidepressant through NMDAR blockade. The relationship between the CRTC1–CREB pathway and agmatine regulation merits further investigation, as it will bring better knowledge of these systems and their contribution to MDD etiology.

## Figures and Tables

**Table 1:** Selection of genes differentially expressed in the cortex of *Crtc1* ^*−/−*^ mice

*Symbol*	*Gene*	*GenBank*	*Fold change*	*FDR* P
*Cartpt*	CART prepropeptide	NM_013732	0.42	0.0086
*Nr4a1*	Nuclear receptor subfamily 4, group A, 1	NM_010444	0.50	0.0181
*Nr4a3*	Nuclear receptor subfamily 4, group A, 3	NM_015743	0.52	0.0103
*Crem*	cAMP responsive element modulator	NM_001110859	0.59	0.0028
*Bdnf*	Brain-derived neurotrophic factor	NM_007540	0.61	0.0131
*Nr4a2*	Nuclear receptor subfamily 4, group A, 2	NM_013613	0.70	0.0476
*Ntrk2*	Neurotrophic tyrosine kinase receptor, 2 (TrkB)	NM_008745	0.81	0.0108
*Agmat*	Agmatine ureohydrolase (agmatinase)	NM_001081408	1.67	0.0099

Abbreviation: FDR *P*, false discovery rate corrected *P*-value.
