# Supplementary material for: Involvement of the agmatinergic system in the depressive-like phenotype of the Crtc1 knockout mouse model of depression
Source: Transl Psychiatry. 2016 Jul 12;6(7):e852–. doi: 10.1038/tp.2016.116 (PMC5545706; doi:10.1038/tp.2016.116)
Supplement: Supplementary Figure 1 [file tp2016116x1.pdf]

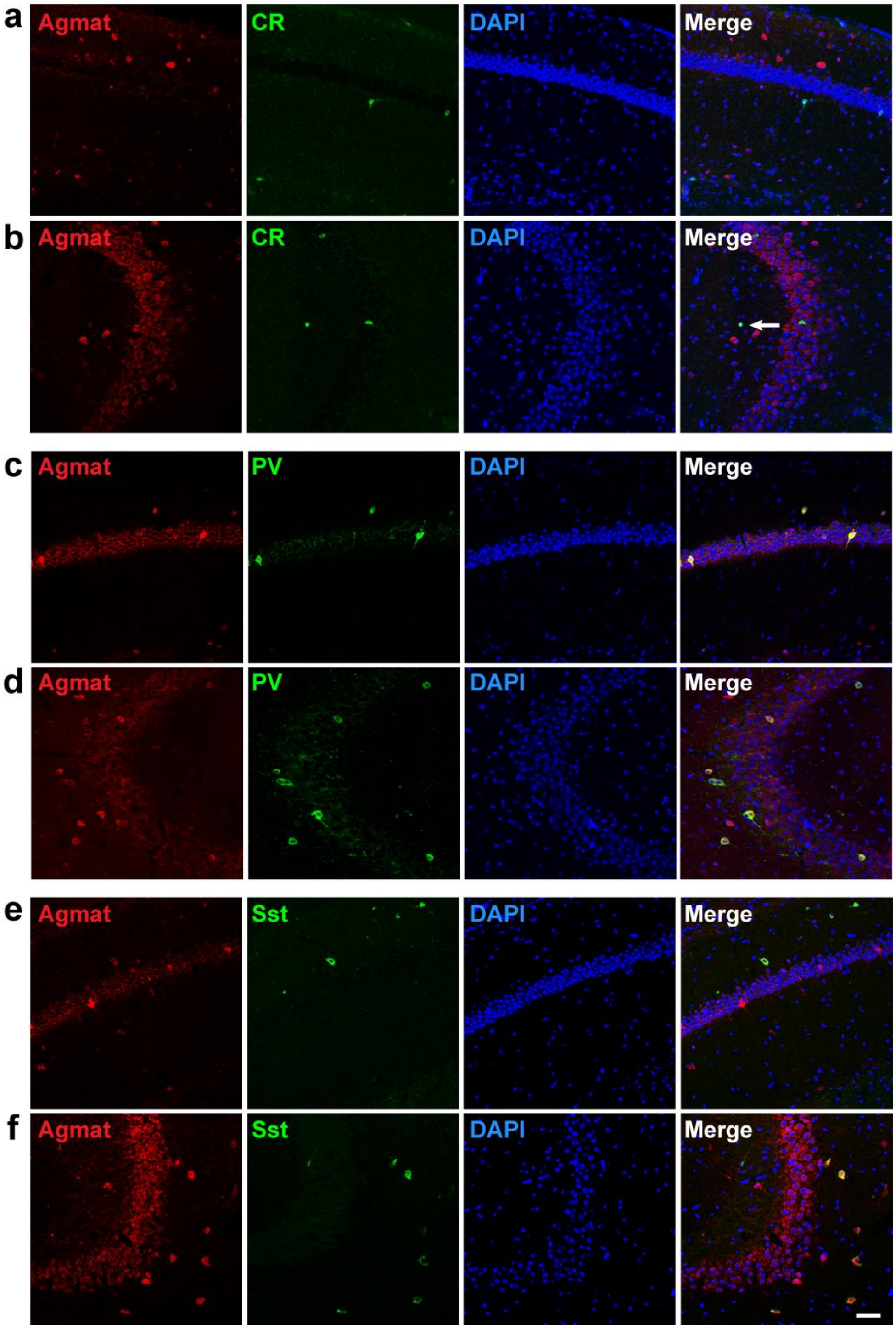

Supplementary Figure S1 Characterization of GABAergic interneurons subpopulations expressing agmatinase (Agmat) in the CA1 and CA3 regions of the hippocampus (HIP) of WT male mice. Double immunofluorescence labeling of (a, b) Agmat and calretinin (CR), (c, d) Agmat and parvalbumin (PV), and (e, f) Agmat and somatostatin (Sst) in WT (a, c, e) CA1 and (b, d, f) CA3 regions of the HIP. Total cells were identified by nuclear DAPI staining. Merged images showed no colocalization of Agmat and CR staining in CA1 (a). Few colocalization could be observed in CA3, as indicated by the arrow. All PV- and most of Sst- interneurons also express Agmat in the CA1 (c, e) and CA3 (d, f) regions of the HIP. Scale bar 50 μm.
