# Supplementary material for: Involvement of the agmatinergic system in the depressive-like phenotype of the Crtc1 knockout mouse model of depression
Source: Transl Psychiatry. 2016 Jul 12;6(7):e852–. doi: 10.1038/tp.2016.116 (PMC5545706; doi:10.1038/tp.2016.116)
Supplement: Supplementary Figure 2 [file tp2016116x2.pdf]

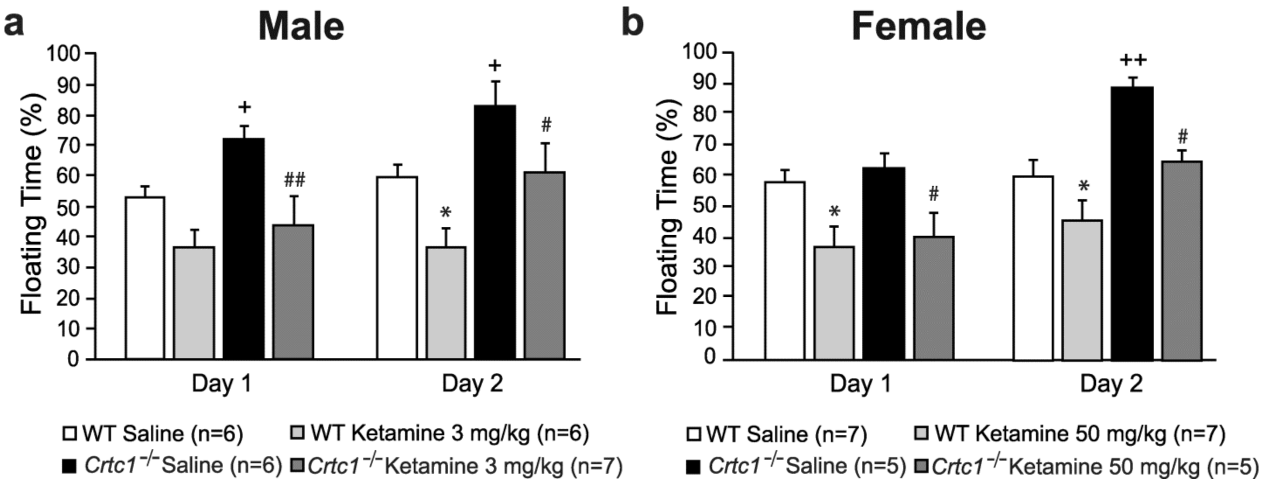

Supplementary Figure S2 Behavioral effects of acute ketamine treatment on male and female WT and *Crtc1*$^{-/-}$ mice. (a-b) Effects of acute ketamine treatment (3 mg/kg, IP) 30 min prior to a forced swim test (FST) during two consecutive days. In male mice (a) vehicle-treated *Crtc1*$^{-/-}$ mice (n=6) showed higher immobility levels than vehicle-treated WT mice (n=6) on both days of test ($^{+}p < 0.05$). Ketamine significantly decreased the immobility time of WT mice ($^{*}p < 0.05$, n=6) on day 1, and *Crtc1*$^{-/-}$ mice ($^{\#}p < 0.05$, $^{\#\#}p < 0.01$, n=7) on both days of test. In female mice (b) ketamine-treated WT and *Crtc1*$^{-/-}$ mice (n=7, n=5 respectively) showed significantly decreased immobility time ($^{*}p < 0.05$, $^{\#}p<0.05$) compared to vehicle-treated WT and *Crtc1*$^{-/-}$ mice (n=7, n=5 respectively) on both days of test. On day 2, vehicle-treated *Crtc1*$^{-/-}$ mice also presented significantly increased immobility time ($^{++}p < 0.01$) than vehicle-treated WT mice. Data are mean ± SEM.
